# Partial siamese twin as potential organ donor

**DOI:** 10.4103/0970-1591.65406

**Published:** 2010

**Authors:** Rakesh Kapoor, Ruchir Maheshwari, Aneesh Srivastava, Raj K. Sharma

**Affiliations:** Department of Urology, Center of Renal Sciences, Sanjay Gandhi Postgraduate Institute of Medical Sciences, Lucknow – 226 014, India; 1 Department of Nephrology, Center of Renal Sciences, Sanjay Gandhi Postgraduate Institute of Medical Sciences, Lucknow – 226 014, India

**Keywords:** HbsAg positive, renal transplant, organ donation, partial Siamese twin

## Abstract

During evaluation of a partial Siamese twin for removal of nonviable parasitic part in an 8-year-old male child, a fully functional kidney was found. The functional status of the extra kidney was found to be within acceptable limits for the purpose of transplant, which was subsequently done in a 24-year-old patient with end-stage renal disease. The recipient is healthy 19 months after the surgery. The possibility of using organs from a partial Siamese twin makes this a unique case report.

## INTRODUCTION

End-stage renal disease (ESRD) affects patients in all age groups and renal transplantation is considered to be the best mode of renal replacement therapy. The treatment is plagued by scarcity of available organs for donation. Live related donor renal transplantation is an acceptable alternative and approved under rule of law of our country. However, there may be nonavailability of blood group compatible donors within the family. The concept of organ donation from brain-dead is yet to be popularized in vast tracts of this country. Partial Siamese twins are a known rarity in medical literature, wherein the nonviable part is usually disposed off or showcased in the shelves of pathology museum. The possibility of utilizing this part as potential organ donor can help in treating multiple patients.

## CASE REPORT

**Figure 1:**
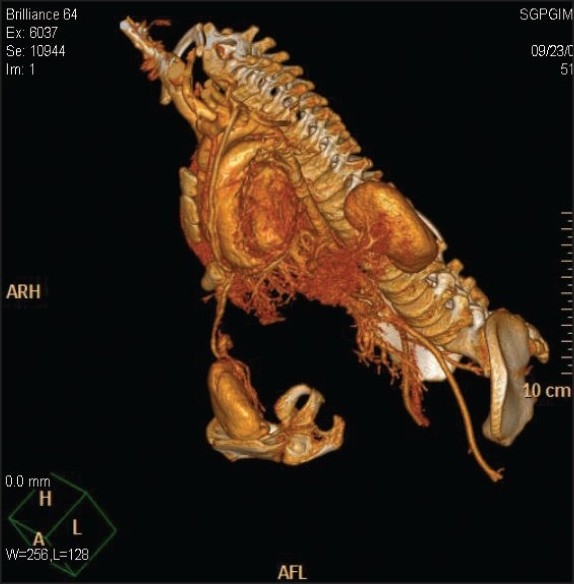
3D-reconstructed CT image showing extra kidney supplied by the internal mammary artery of child

An eight-year-old partial Siamese twin presented for evaluation to eliminate the nonviable part. During evaluation, an extra fully functional pancake-shaped kidney was found [Figure 1]. On detailed functional assessment, the extra kidney was found to have a glomerular filtration rate (GFR) of 40 ml/min/1.73 m^2^; body surface area (BSA). The kidney was supplied by multiple arteries arising from the main vessel of the partial Siamese twin, which was a direct continuation of internal mammary artery of the child. This kidney was draining by means of two separate ureters into a separate bladder, which was emptied by a fully developed urethra and penis of the partial Siamese child. However, the child was also found to have HbsAg-positive status. Therefore, an HbsAg-positive potential recipient was searched for.

The recipient was a 24-year-old man with ESRD, diagnosed incidentally for evaluation of chest pain and hypertension, with basic disease chronic interstitial nephritis. No blood group–compatible donors were available in the immediate family. Hence, the patient was kept on maintenance hemodialysis, with native urine output ≈600 ml and baseline serum creatinine ≈4.7 mg/dl. Patient contacted Hepatitis B virus infection while on maintenance hemodialysis and was started on tablet Telbivudine and showed favorable response.

With profiles matching favorably, cross-matching was done, and was negative at all temperatures. The decision was taken to perform transplant kidney after due consultation with the institute's ethical committee. The prospective recipient and his relatives were explained in detail about the uniqueness of the case, with no previous case reports of this nature, and a fully explained consent was obtained, including the risk of graft nephrectomy. After this, recipient was started on a D–2 triple drug immunosuppression induction protocol (tacrolimus, mycophenolate mofetil and prednisolone).

**Figure 2:**
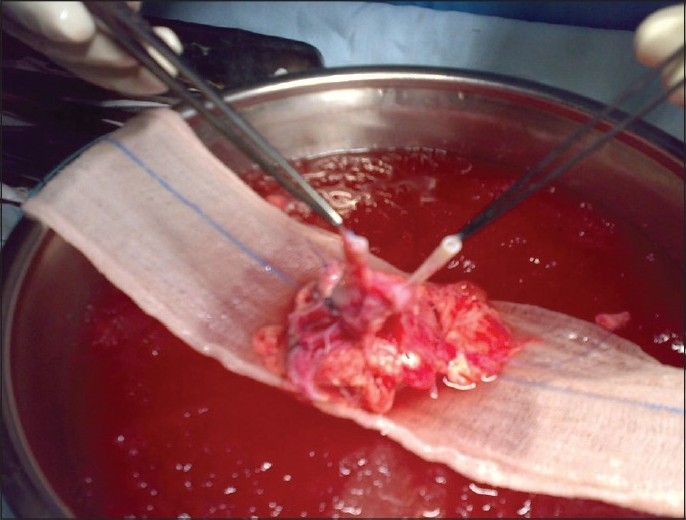
Kidney graft showing two ureters

Organ retrieval was carried out at a private hospital that catered to plastic surgery. The internal mammary artery and vein were acting as the aorta and inferior vena cava of the Siamese twin, supplying upper limbs, multiple branches to pancake kidney and finally supplying both lower limbs. The main vessels were isolated, with adequate length and after control from the origin; the artery and vein were ligated infrarenally while the upper ends were cut as in normal transplant kidney for cooling. Both the ureter and cuff of bladder were removed with the pancake kidney with adequate margins [Figure 2]. The kidney was perfused with chilled Eurocollins solution, packed in a polybag in a container with ice sludge, and was expediently shifted in an icebox to the SGPGI (within 20 min). After retrieving the kidney, the remaining organs of the partial Siamese twin were removed and the abdominal wall closed by another plastic surgery team.

In urology OT, bench surgery was performed to join together the ureters in a Wallace-like fashion. The kidney was placed in renal bed prepared in the right iliac fossa extraperitoneal space and donor artery and vein were anastomosed to the internal iliac artery and external iliac vein, respectively. The ureteroneocystostomy was done by modified Leich-Greigor method with bilateral double-J stents.

Postoperatively after initial dieresis, the patient went into oliguria; diagnosis of an acute tubular necrosis was made and treated conservatively. After around two months, the donor kidney gradually picked up function and serum creatinine stabilized to around 1.2 mg/dl.

Presently, 19 months since transplant, recipient is doing fine with s.creatinine stablised at 1.4 mg/dl.

## DISCUSSION

Cadaveric and live related donor renal transplantations are well established and the programs are running successfully in many centers worldwide. However, due to ever increasing number of ESRD patients and constant shortage of organs, there is ever inflating waiting list in all high-volume centres. Tissue engineering is still in infancy stage and a distant dream to create new kidneys for these patients. To increase the woeful paucity of organs, the eligibility criteria of donors has been relaxed to include more people, previously excluded, termed as the expanded criteria or marginal donors. Pediatric donors have been accepted as potential cadaveric donors and few series addressing to this issue have been published, showing both short- and mid-term results. Foss *et al*, have published a study of 19 recipients who were followed up for 5–9 years, with 8 having donors aged < 8 years. They have emphasized the use of both kidneys en bloc when using kidneys from donors aged below 5 years.[1] Similarly, Csapo *et al*, have published similar recipient outcomes when comparing ideal adult vs. pediatric donors.[2] Ahn *et al*, have recommended that in the present era of modern antiviral agents, such as lamivudine, renal transplantation in HbsAg-positive ESRD patients should not abandoned.[3] Veruox *et al*, have also recommended that kidneys from HbsAg-positive donors can be safely transplanted to HbsAg-seropositive recipients.[4] Thomson *et al*, have published that horseshoe kidneys from pediatric and adult donors can be safely transplanted into adult recipients.[5] However, there is no reported case in English literature about organ retrieval from a partial Siamese twin, which we believe is the first ever case in the world. Although in the present case, only kidney has been retrieved and transplanted, there is a definitive possibility of use of other retrievable organs for use in multiple donors if suitable patients can be found.
